# A spatiotemporal analysis of opioid prescriptions in Indiana from 2015 to 2019

**DOI:** 10.1186/s13011-025-00664-8

**Published:** 2025-08-08

**Authors:** Paula A. Jaimes-Buitron, Nicole Adams, Nan Kong, Carolina Vivas-Valencia

**Affiliations:** 1 Department of Biomedical Engineering and Chemical Engineering, The University of Texas at San Antonio, San Antonio, TX USA; 2 School of Nursing, Purdue University, West Lafayette, IN USA; 3 Weldon School of Biomedical Engineering, Purdue University, West Lafayette, IN USA; 4 Edwardson School of Industrial Engineering, Purdue University, West Lafayette, IN USA

**Keywords:** Opioid prescriptions, Opioid prescribing rates, Moran's I, Indiana, Geospatial analysis, Urbanicity

## Abstract

People living in rural communities are more likely to receive opioid prescriptions, partly due to job-related injuries. State-level interventions have reduced opioid prescribing; however, rural/urban disparities persist due to differences in demographics and prescribing practices, particularly in states with large rural populations like Indiana. While spatiotemporal analyses have explored aspects of the opioid crisis, spatiotemporal patterns of opioid prescribing have not been sufficiently studied. This study utilizes a sample of Medicaid claims data from the Indiana Family and Social Services Administration from 2015 to 2019 to analyze spatiotemporal patterns of patients receiving at least one opioid prescription across Indiana. The goal was to analyze patient demographics and track prescription hotspot movement over time in rural and urban areas. We analyzed data for 107,574 Medicaid enrollees who received opioid prescriptions during the study period. We found that most patients in the cohort resided in urban areas, with the number of patients who were prescribed opioids and resided in rural areas decreasing at a faster rate. We conducted a negative binomial regression analysis to examine the relationship between various demographics (sex, age, race/ethnicity, and urban/rural classification) and the number of patients receiving at least one opioid prescription over time. Our findings indicate that older patients, patients identifying as females, patients who identify as White, and patients living in urban areas, are more likely to receive at least one opioid prescription. Additionally, the interaction effects revealed that patients from all demographic groups were more likely to receive at least one opioid prescription if they lived in urban areas, compared to those living in rural areas. Using Local Moran’s I as a local spatial autocorrelation measure, we identified clusters highlighting transitions from rural to urban areas over time. In 2015–2016, three significant clusters emerged within rural-surrounded 3-digit ZIP codes (472, 474, 476), based on the Rural-Urban Commuting Area Codes. Over time, significant clusters shifted towards urban or mixed areas, possibly influenced by state guidelines and legislation. These findings enhance the understanding of opioid prescription dynamics and identify patterns in opioid prescribing rates in terms of the proportion of patients receiving opioid prescriptions among urban vs. rural communities in Indiana.

**Supplementary Information:**

The online version contains supplementary material available at 10.1186/s13011-025-00664-8.

## Background

The opioid crisis remains a critical public health issue in the United States, marked by widespread misuse of both legal and illegal opioids [1]. In 2021, 3.3% of people aged 12 and older reported misuse of prescribed or illicit opioids in the past year [2]. During the same period of time, 15.7% of all opioid overdose deaths in the United States were related to opioid prescriptions [3]. Reductions in opioid prescribing have been observed following state-level regulatory interventions [4], however, certain areas continue to exhibit disproportionately high opioid prescribing patterns, which may indicate outliers or pockets where prescriptions remain elevated despite broader efforts to reduce unnecessary prescribing [5].

The prescription rates for opioids differ between rural and urban areas, with rural areas showing higher rates of opioid prescriptions [6–8]. Individuals living in rural communities are more likely to receive and use prescribed opioids due to job-related injuries requiring pain management [9]. Moreover, non-metropolitan areas with larger populations of older adults tend to have higher prescription rates due to the increased prevalence of pain-related conditions [6, 10]. In Indiana, where 21.9% of the population lives in non-metropolitan areas [11], opioid prescription misuse has been reported at a prevalence of 3.8% [12]. In remote areas where the distances to hospitals or healthcare facilities were often long, patients have historically faced challenges in accessing care, often making infrequent day-long trips to see a physician. As a result, many physicians prescribed larger amounts of opioids during one visit to optimize their patients’ time, although this practice has been regulated in recent years [13]. Overprescribing could potentially result in an excess of opioid analgesics being stored by patients, which could then be accessed by relatives or friends for misuse [2, 10].

Geographic disparities play a critical role in understanding the opioid crisis, as socioeconomic conditions, healthcare access, and local policies can vary significantly across regions. Prior research has implemented spatiotemporal analysis to identify these disparities contributing to the opioid crisis, such as the diagnosis of opioid use disorder (OUD) and opioid overdose-related mortality [14–18]. A study conducted in 2020, analyzed spatiotemporal patterns in Ohio, revealing that clusters of increased overdose deaths were primarily located in economically vulnerable areas characterized by limited job and educational opportunities [14]. Another study from 2021 used Moran’s statistics and Bayesian multivariate spatiotemporal modeling to analyze opioid overdose death hotspots and mortality rates in Ohio [15, 16]. The study revealed a higher concentration of opioid overdose deaths in urban areas compared to rural areas in Ohio over time. It was also observed that the illicit fentanyl market experienced rapid growth in urban cities, contributing to a surge in overdoses in recent years [16]. Additional applications of spatiotemporal analysis have also been used in multi-state analyses of opioid prescribing patterns [17] and to analyze the trends in overdose-related deaths at the county level nationwide [18]. However, in Indiana, spatiotemporal analyses of the opioid crisis have been limited, particularly in understanding the dynamics of opioid prescribing patterns in rural vs. urban areas. The studies mentioned above have successfully used spatiotemporal analysis to identify patterns in opioid-related outcomes, such as overdose hotspots and rates of diagnosed OUD, suggesting that similar methods could be effectively applied to analyze opioid prescribing patterns. This should account for both the spatial distribution of individuals receiving opioid prescriptions and the temporal changes, which are crucial for understanding evolving prescribing patterns in rural and urban areas. Understanding these dynamics at the geographic level provides valuable evidence to guide resource allocation and inform policymakers in developing effective interventions.

In this study, we conducted a spatiotemporal assessment of opioid prescriptions and their trends in rural and urban areas of Indiana. We focused on Indiana for two main reasons: first, Indiana has been disproportionately affected by the opioid crisis, with a high rate of opioid prescriptions contributing to significant public health challenges [19]. Second, the state’s mix of rural and urban communities provides a unique opportunity to study how geographic dynamics shape opioid prescribing patterns [20]. The goals of the study were to (1) identify spatiotemporal variations in opioid prescribing rates among Medicaid enrollees from 2015 to 2019 in Indiana, focusing on the number of patients receiving at least one opioid prescription, (2) assess the clustering of opioid prescribing rates in urban and rural areas, and (3) identify differences in opioid prescribing among various population subgroups within the Medicaid cohort. Medicaid is the primary public health insurance program in the U.S. for individuals with low income, covering a significant portion of those affected by OUD. Approximately 40% of nonelderly adults diagnosed with OUD are Medicaid beneficiaries [21]. The program also serves a diverse population with varying health conditions, including chronic illnesses and mental health disorders [22]. Given Medicaid’s role in providing healthcare access to vulnerable populations, analyzing its claims data allows for a comprehensive assessment of opioid prescribing patterns among patients with a higher likelihood of receiving opioid prescriptions. We anticipate that our methodological framework, which incorporates techniques from data mining and geographic information systems (GIS), can be extended for future analyses using similar geographic data. Additionally, it may assist in the development of new predictive models and support further evaluations, such as refined assessments of geographic trends or the identification of new patterns in healthcare data.

## Methods

### Study design and data population

In this study, we used a sample of the reimbursement claims database from Indiana Medicaid provided by the Indiana Family and Social Services Administration (FSSA) covering the period from January 2015 to December 2019. The study was approved by the Purdue University Institutional Review Board (2019–118). The dataset includes patient demographics such as age, sex, race/ethnicity, and geographic information, which is limited to 3-digit ZIP codes. Additionally, the dataset contains information about prescribed medications, identified using the National Drug Code (NDC) directory, Billing National Provider Identifiers (NPI), and the service date. Details about prescription information, including dosages and quantities, are unavailable and, therefore, not included in this study.

**Fig. 1:**
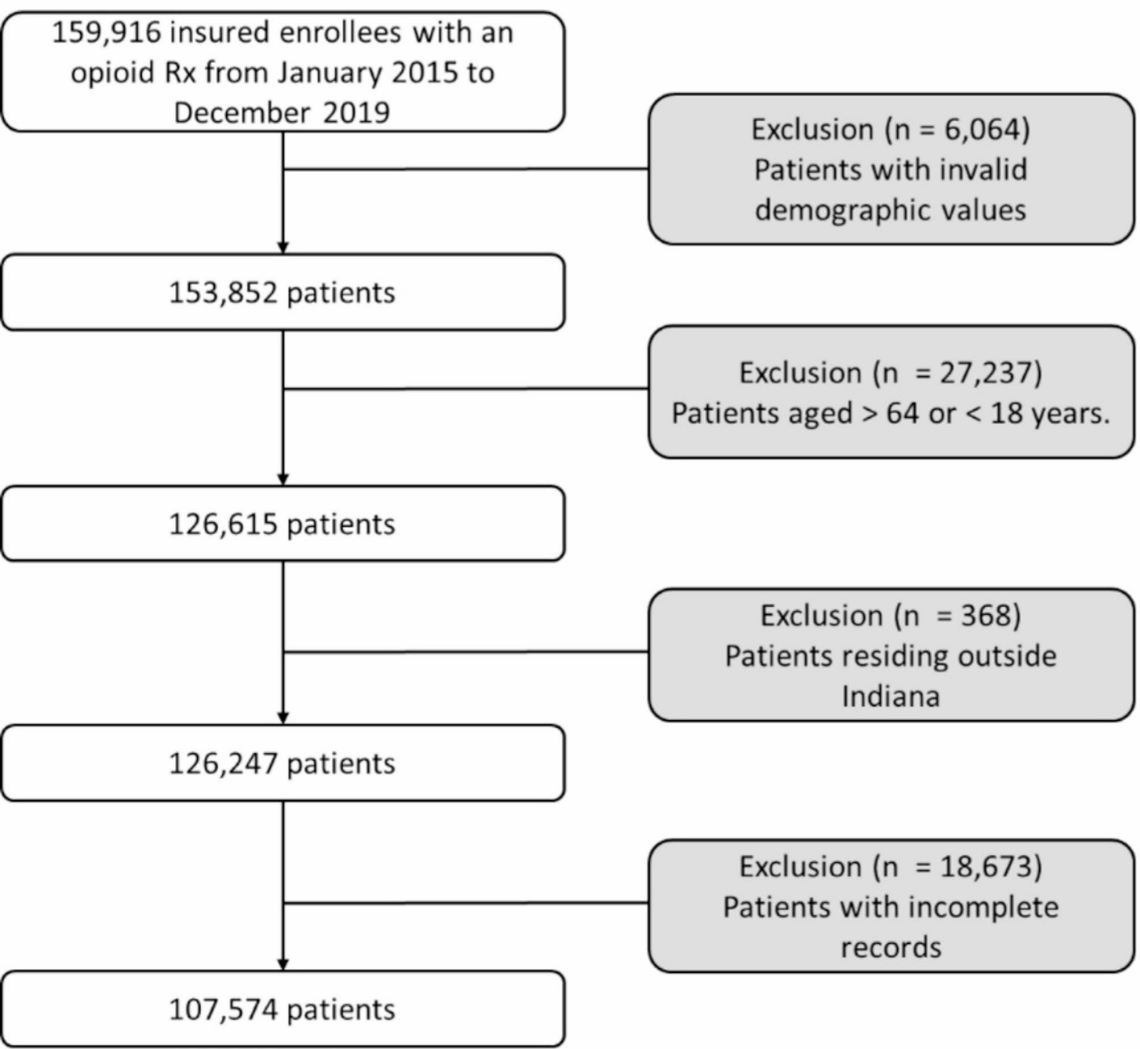
Workflow for extraction of the cohort

**Fig. 2:**
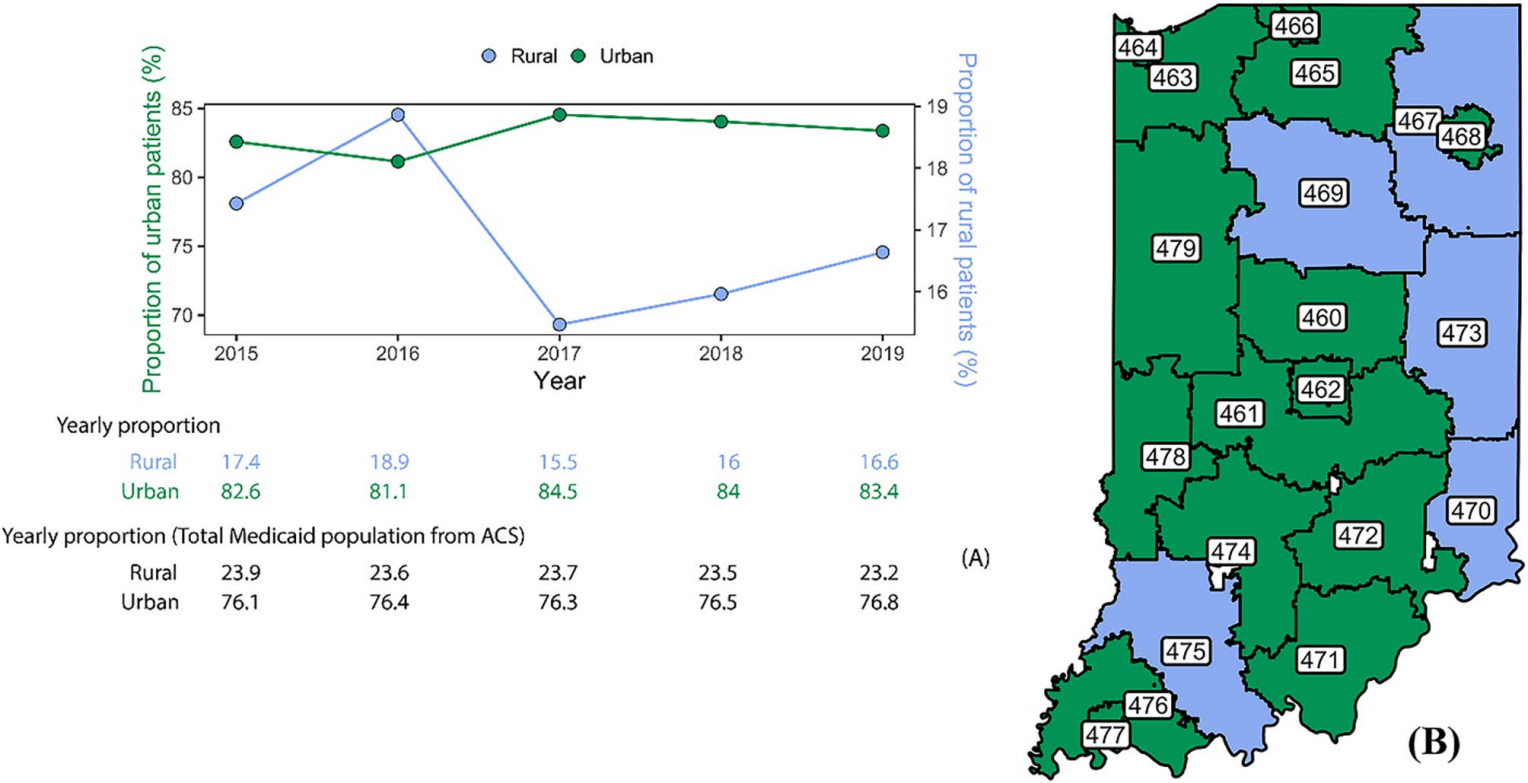
Classification of patients receiving at least one opioid prescription by rural and urban areas in Indiana. **A**). Proportion of patients receiving an opioid prescription by rural and urban classification from the FSSA database and total Indiana Medicaid population from ACS. **B**). Localization of the 3-digit ZIP codes and the classification in rural and urban areas. Green 3-digit ZIP codes are classified as “Urban” and blue 3-digit ZIP codes are classified as “Rural”. The urban/rural classification was extracted from RUCA Codes and transferred to a ZIP code approximation. To obtain the classification by 3-digit ZIP codes, a weighted average was calculated using the average of the RUCA codes by 5-digit ZIP codes and the total estimate of the population by each 5-digit ZIP code aggregated by the first 3-digit ZIP codes. The RUCA Codes encompass a number from 1 to 10 for the primary classification and a decimal part to indicate if there are other connections between rural and urban areas. Primary RUCA Codes are classified as isolated (RUCA 10), small town (RUCA 7–9), micropolitan (RUCA 4–6), or metropolitan (RUCA 1–3). RUCA 4 is a micropolitan area core with a primary flow within an urban cluster of 10,000 to 49,999 (large UC), so we considered RUCA 4–10 as “rural” and RUCA 1–3 as “urban”. Mann-Whitney U test between rural and urban population from our cohort and Indiana Medicaid from the ACS showed no significant differences (*p*-value > 0.05)

We identified patients who received at least one opioid prescription using the NDC identifier between 2015 and 2019. The complete list of opioid prescriptions is in Supplementary Table S1. Patients were counted only once per year if they received at least one opioid prescription. However, a single patient could appear in multiple years if they continued to receive opioid prescriptions over time. Because our analysis focuses on the number of patients receiving opioid prescriptions rather than the number of prescriptions filled, we did not track multiple prescriptions per patient within the same year. We excluded claims if they lacked information on the medication prescribed or demographic variables. We also excluded patients not residing in Indiana, based on the 3-digit ZIP code provided. We included patients aged 18 to 64 years relative to each year (See Fig. 1). We identified if the patient resided in a rural or urban area using the Rural-Urban Commuting Area (RUCA) Codes converted to a ZIP code approximation [23]. RUCA Codes classify census tracts and ZIP code approximations into rural and urban using measures of population, density, urbanization, and commuting [23]. To obtain the RUCA code for each 3-digit ZIP code, we calculated a weighted average using the population of each 5-digit ZIP code provided by American Community Survey (ACS) data, which contains population and housing estimates by different geographic levels. We aggregated the estimates for each 5-digit ZIP code by their first 3-digit ZIP code [24, 25] (See Fig. 2B). Opioid prescribing rates were calculated by dividing the number of patients who received an opioid prescription by the total Medicaid population in Indiana, specifically those aged 18 to 64 years, as reported by the ACS. To assess disparities among demographic groups, such as sex, age groups, race/ethnicity, and urban/rural classification, we employed a negative binomial regression [26]. This model enables us to evaluate how multiple demographic variables, including age, race/ethnicity, sex, and rural/urban classification, interacted with the number of patients receiving at least one opioid prescription from 2015 to 2019. The negative binomial model is a generalization of the Poisson regression model, allowing us to better model count data by incorporating an additional parameter to address overdispersion. The significance level for the negative binomial regression model was set at a p-value < 0.05. 

### Spatiotemporal pattern analysis at 3-ZIP codes level to discover potential global and local spatial clusters

Our database includes Medicaid enrollees who received an opioid prescription between 2015 and 2019, but we do not have access to the entire Medicaid population. In order to be able to determine the proportion of Medicaid enrollees who received an opioid prescription, we normalized the total number of patients receiving an opioid prescription in each 3-digit ZIP code by dividing the number of patients receiving a prescription by the aggregated Medicaid enrollees count in Indiana for each respective 3-digit ZIP code, provided by the ACS. To determine spatial patterns of opioid prescribing, we used spatial autocorrelation statistics to identify if patients receiving an opioid prescription tend to be concentrated or dispersed in a specific region. We used Moran’s I statistics (Global and Local) widely used measures of spatial autocorrelation [15, 27, 28]. Global Moran’s I was utilized to test for the overall presence of spatial clusters without identifying their specific locations. It ranges from + 1 (strong positive spatial autocorrelation, where similar values cluster together) to -1 (strong negative spatial autocorrelation, where dissimilar values cluster), with 0 indicating a random spatial pattern. Permutation was utilized to calculate the reference distribution for Moran’s statistic to assess the null hypothesis of spatial randomness. Then we applied Local Indicators of Spatial Autocorrelation (LISA) [29] to identify the locations of these hotspots, i.e., the core or centers of the concentrated areas with a high rate of patients receiving opioid prescriptions, and their statistical significance. Local Moran’s I follow four statistics: High-High, High-Low, Low–High, and Low-Low. The first designation (e.g., High-) specifies the degree of LISA statistics within each 3-digit ZIP code, while the second designation (e.g., -Low) indicates the level of LISA for neighboring regions in terms of contiguity. For example, a High-Low classification for a 3-digit ZIP code means that the LISA statistic of the region is significantly high, but the values of the surrounding neighbors are significantly low. It is important to note that these classifications reflect spatial relationships, not absolute prescribing rates or their changes over time. For example, a High-High designation does not imply increasing opioid prescribing rates; it only indicates clustering of similar high values. Similarly, a High-Low or Low-High label identifies areas that differ significantly from their neighbors. The spatial autocorrelation analysis was carried out using GeoDa version 0.0.10-4 [30], involving a significance level set at a p-value <0.05.

### Evaluating the modifiable areal unit problem in spatiotemporal analysis

The Modifiable Areal Unit Problem (MAUP) arises when spatial analyses depend on the chosen geographic aggregation level, potentially changing statistical relationships and spatial patterns [31]. To determine whether the spatial aggregation of the percentage of patients who received at least one opioid prescription at the 3-digit ZIP code level affects the findings, we transformed geographic information into county-level data. This was done using crosswalk tables provided by the U.S. Department of Housing and Urban Development (HUD) [32]. These crosswalk tables facilitate spatial disaggregation by identifying the proportion of each ZIP code’s population that resides in each county. We began by multiplying the number of patients prescribed at least one opioid prescription by their respective residential ratios from the crosswalk table. This table indicates the proportion of each ZIP code’s population that belongs to each county. This process allows us to create a weighted redistribution of the number of patients prescribed at least one opioid prescription, which is then aggregated at the county level by summing the results across all the 3-digit ZIP codes within each county. Next, we similarly redistributed the total Medicaid population from ACS at the 3-digit ZIP code level to compute the final county-level proportion of patients prescribed at least one opioid prescription. To determine whether spatial aggregation influenced statistical relationships, we conducted a bivariate ordinary least squares (OLS) regression analysis at both the 3-digit ZIP code level and county level. This model measures the relationship between year and the proportion of patients receiving at least one opioid prescription per spatial unit [31]. We then evaluated whether regression coefficients and model fit $\:{\text{R}}^{2}$ were the same across 3-digit ZIP codes and county level, or the aggregation introduced distortions due to MAUP effects. To test the normality and the stationarity of the error for both models, we ran the Jarque-Bera and Breusch-Pagan tests [33, 34]. Finally, we calculated the Local Moran’s I at the county level and compared it with the results from the 3-digit ZIP level. This comparison allowed us to determine whether spatial aggregation affected the statistical significance of clusters or introduced artificial patterns due to the redistribution effects.

## Results

### Demographic-based analysis across years

In our cohort, 107,574 out of 156,916 Medicaid enrollees in the FSSA database received at least one opioid prescription between January 2015 and December 2019. Of all the patients, 72,181 (67.10%) identified as females (See Table 1). The majority of the patients (76.30%) reported White as their respective race/ethnicity, followed by Black (17.95%), Hispanic (4.43%), and other races (1.32%). The percentage of patients prescribed opioids remained consistent across age groups from 2015 to 2019, with the largest number of prescriptions written for those in the 26 to 54 age group. The proportion of patients receiving at least one opioid prescription residing in urban areas increased by 0.97% from 2015 to 2019, while the proportion in rural areas decreased by 4.60% during the same time window (See Fig. 2A). Year by year, the number of patients residing in urban areas decreased by 1.82% (2015–2016), followed by an increase of 4.19% (2016–2017), then a small decrease of 0.6% (2017–2018), and finally another decrease of 0.71% (2018–2019). For the patients residing in rural areas, there was an initial increase of 8.62% (2015–2016), with a subsequent decrease of 17.99% (2016–2017), followed by an increase of 3.75% (2017–2018), and finally an increase of 3.75% (2018–2019).

**Table 1:** Demographic estimations of patients receiving at least one opioid prescription by year from the study cohort

Characteristics	No. (%) of patients*					*p*-value**
	2015 (*n* = 34,889)	2016 (*n* = 46,571)	2017 (*n* = 17,134)	2018 (*n* = 20,274)	2019 (*n* = 13,441)	
**Sex**						
Female	24,214 (69.4)	31,465 (67.6)	11,628 (67.9)	13,719 (67.7)	8,985 (66.8)	***
Male	10,675 (30.6)	15,106 (32.4)	5,506 (32.1)	6,555 (32.3)	4,456 (33.2)	***
**Race**						
White	26,831 (76.9)	35,943 (77.2)	12,832 (74.9)	15,261 (75.3)	10,477 (77.9)	***
Black	6,475 (18.6)	8,199 (17.6)	3,209 (18.7)	3,808 (18.8)	2,189 (16.3)	***
Hispanic	1,367 (3.9)	2,073 (4.5)	718 (4.2)	863 (4.3)	525 (3.9)	0.00172
Other	216 (0.6)	356 (0.8)	375 (2.2)	342 (1.7)	250 (1.9)	***
**Age**						
18 to 25	4,146 (11.9)	5,632 (12.1)	2,110 (12.3)	2,136 (10.5)	1,239 (9.2)	***
26 to 34	6,736 (19.3)	9,463 (20.3)	4,101 (23.9)	4,379 (21.6)	2,773 (20.6)	***
35 to 44	7,857 (22.5)	10,387 (22.3)	4,080 (23.8)	4,873 (24.0)	3,179 (23.7)	***
45 to 54	8,812 (25.3)	11,527 (24.8)	3,898 (22.8)	4,798 (23.7)	3,312 (24.6)	***
55 to 64	7,338 (21.0)	9,562 (20.5)	2,945 (17.2)	4,088 (20.2)	2,938 (21.9)	***

* Based on patients from Indiana Medicaid in the FSSA database who received at least one opioid prescription

** A Pearson’s Chi-squared test was used to assess the relationship between the demographic groups across years

*** A significant difference was set at *p*-value < 0.001

We conducted a negative binomial regression analysis to investigate the relationship between the number of patients receiving at least one opioid prescription and key demographic factors including age, race/ethnicity, sex and rural/urban status, over time. The model included main effects, two-way and three-way interactions, which evaluates how one or more demographic variables interact over time. The results from the binomial regression model are reported in Supplementary Material Table S2.

The analysis revealed a significant overall decline in the number of patients receiving at least one opioid prescription over time (Rate Ratio [RR]: 0.703, p-value < 0.05). When examining opioid prescription patterns based on urban or rural classification, it was found that patients living in rural areas were less likely to receive an opioid prescription (RR: 0.249, p-value < 0.05). In terms of age, older age groups were more likely to receive at least one opioid prescription compared to patients aged 18–25. Patients aged 26 to 43 were 45.3% more likely to receive an opioid prescription (RR: 1.453, p-value < 0.05), while those aged 45 to 54 had the highest likelihood (RR: 1.830, p-value < 0.05). Racial/ethnic disparities were also evident in the data. Black patients were 61.1% less likely to receive an opioid prescription compared to White patients (RR: 0.389, p-value < 0.05), and patients classified as other races had the lowest likelihood (RR: 0.009, p-value < 0.05). Although Hispanic patients were less likely to be prescribed at least one opioid prescription compared to White patients, this relationship was not statistically significant (RR: 0.986, p-value = 0.882). Sex disparities were also observed, with male patients being significantly less likely to receive an opioid prescription compared to female patients (RR: 0.277, p-value< 0.05). Furthermore, patients in rural areas had significantly lower rates of opioid prescription than those in urban areas (RR: 0.249, p-value< 0.001), indicating that patients living in rural areas were 75.1% less likely to receive opioid prescriptions than their urban counterparts.

Several interaction effects reveal notable differences in prescribing patterns for opioid prescriptions. The interaction between year and race/ethnicity (RR: 1.353,  p-value< 0.001) indicates that the decline in opioid prescriptions over time has been less pronounced for patients classified as other races compared to White patients. Furthermore, age and race/ethnicity interactions show that older Black patients were less likely to receive an opioid prescription than their younger counterparts, particularly in the 35–44 age group (RR: 0.689, p-value=  0.008) and 45–54 age group (RR: 0.720,  p-value= 0.018). Similarly, Hispanic patients aged 55–64 were less likely to receive an opioid prescription compared to their younger counterparts (RR: 0.518,  p-value< 0.001).

Rural disparities in opioid prescriptions also varied across racial/ethnic groups. Black patients in rural areas were significantly less likely to receive an opioid prescription compared to those in urban areas (RR: 0.196,  p-value< 0.001). Hispanic patients in rural areas also were less likely to receive an opioid prescription (RR: 0.316,  p-value< 0.001). Moreover, patients classified as other races in rural areas experienced a 57.4% reduction in opioid prescription rate compared to their urban counterparts (RR: 0.426,  p-value< 0.001). Finally, sex differences in opioid prescriptions were moderated by age. Older male patients, specifically aged 45–54 and 55–64, had significantly higher rates of opioid prescription compared to younger males (RR: 1.761,  p-value= 0.003 and RR: 1.811, p-value< 0.001, respectively). This suggests that sex disparities in opioid prescriptions diminish as age increases.

### Global and local spatial clusters of opioid prescribing using spatiotemporal analysis at 3-digit ZIP code level

**Fig. 3:**
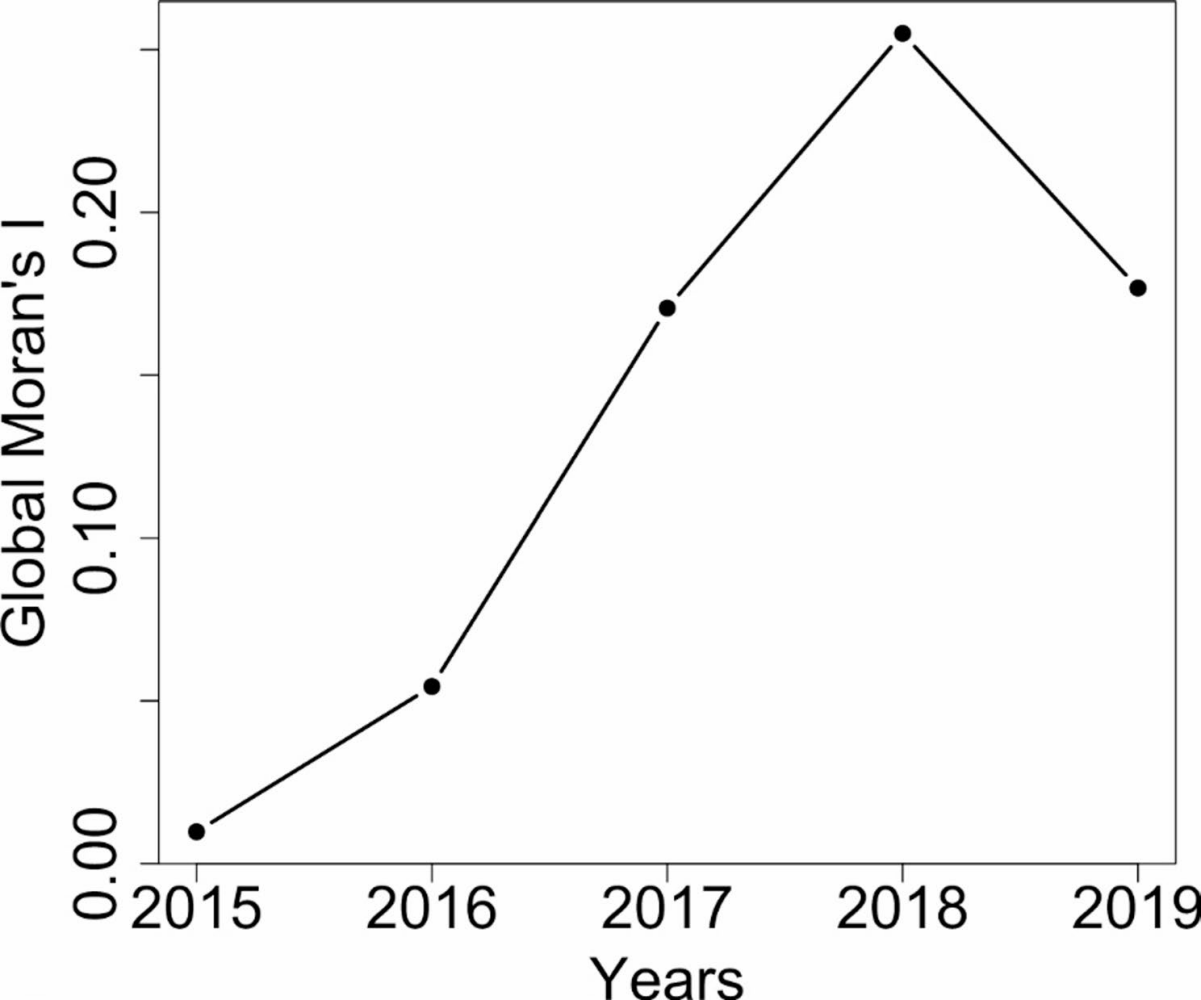
Global Moran’s I statistics across 2015 to 2019. Global Moran’s I determine the overall spatial concentration of the proportion of patients receiving at least one opioid prescription in Indiana. A higher value for Global Moran’s I indicate highly concentrated areas. Global Moran’s I indicate the presence of spatial concentration but not the locations for the spots

Global Moran’s I was utilized to identify if clusters existed along regions without pinpointing the locations of those clusters identified. From 2015 to 2018, there was an increase in the Global Moran’s I value, indicating that opioid prescribing clusters became more spatially concentrated during this period (See Fig. 3). However, there was a decrease observed in 2019, indicating that the distribution of patients receiving an opioid prescription was spread across regions in Indiana. Our analysis revealed significant Global Moran’s I values for 2018 and 2019 (p-value < 0.05). This indicates a high spatial concentration of patients receiving opioid prescriptions from 2018 to 2019. Refer to Supplementary Material Table S3 for detailed values for each year.

**Fig. 4:**
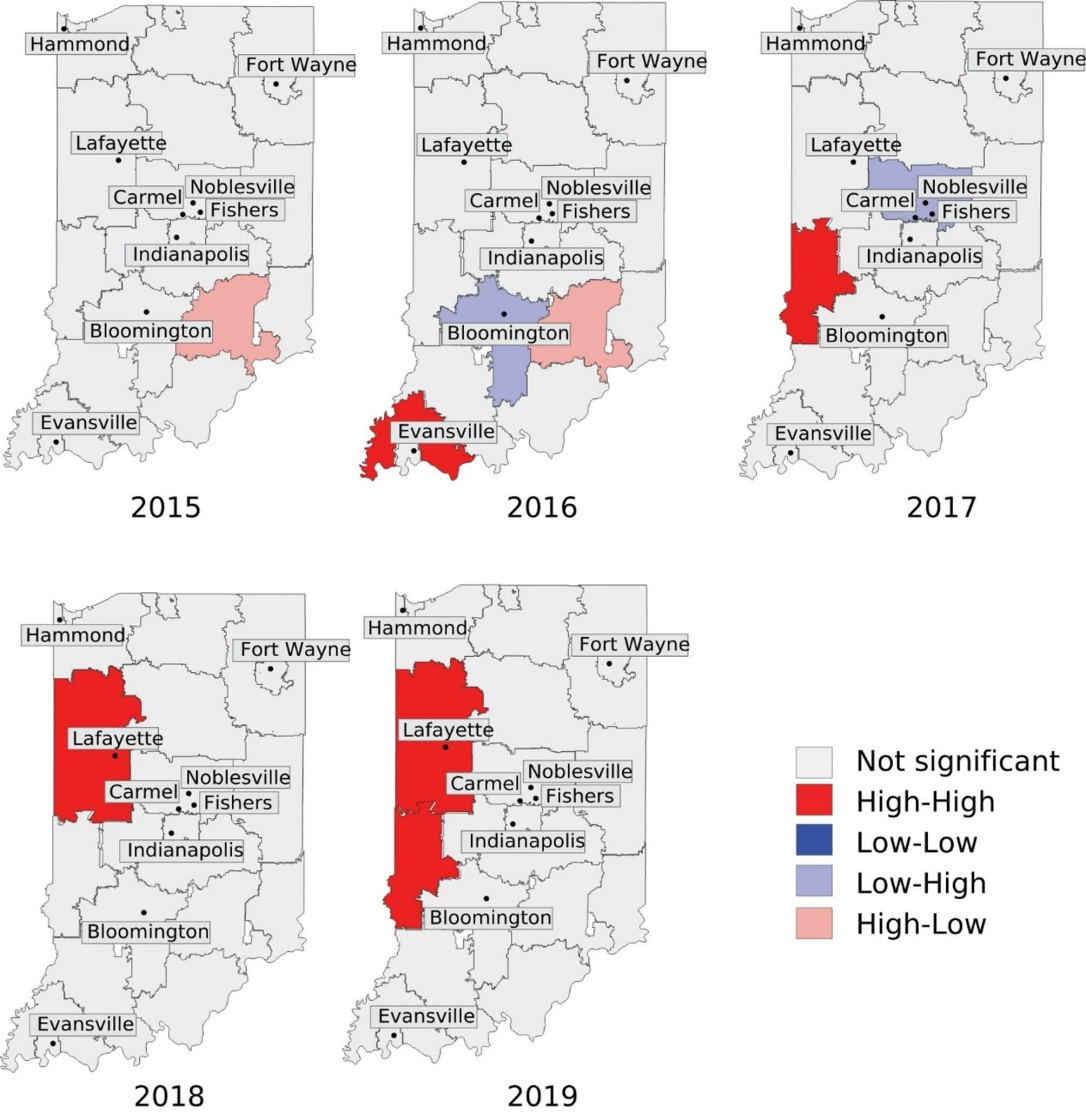
Spatial clusters with normalized patients receiving an opioid prescription by 3-digit ZIP codes, Indiana, 2015–2019. Local Moran’s I identify the specific location of the core clusters or centers of the agglomerated zones of patients receiving an opioid prescription. There are four statistics of LISA, where the first field of each of those refers to the level of proportion of patients in the core or center of the cluster and the second field indicates the level of proportion of patients in surrounding zones. High-High label means that the core and the neighbors have a high number of patients receiving opioid prescriptions. High-low and Low-high are called outliers and describe the behavior of having a core with a different number of patients in comparison with their surrounding neighbors. The significance level is calculated under the null hypothesis of random spatial distribution with a p-value < 0.05. The names of the cities presented in the figure are the 10 most populated cities in Indiana

Figure 4 shows the outcomes of Local Moran’s I from 2015 to 2019, which was used to identify the locations of opioid prescription concentrated regions. Initially, we observed a core cluster in the 472 3-digit ZIP code near suburban areas, and also rural areas (See Fig. 2B), with a High-Low rating in 2015. In 2015, the 472 3-digit ZIP code had an opioid prescription rate of 228 per 1,000 population. This area was surrounded by neighboring 3-digit ZIP codes that reported significantly lower rates; for example, the 470 3-digit ZIP code had a prescription rate as low as 42 prescriptions per 1,000 population (See Supplementary Material Table S6). In 2016, a High-High core cluster was identified in the 476 3-digit ZIP codes, surrounded by 475 3-digit ZIP codes classified as rural (See Supplementary Material Table S4 and Table S5). This cluster corresponds with the highest rates of opioid prescription for that year, which ranged from 335 to 391 per 1,000 population (See Supplementary Material Table S6). Over time, clusters have become more prevalent in urban areas. In 2017, one significant core cluster (478 3-digit ZIP codes) was identified as surrounded by mostly urban areas, particularly the 461 and 479 3-digit ZIP codes. These areas had opioid prescription rates of 137.94 and 140.70 per 1,000 population, respectively; on the other hand, the core clusters in the 460 3-digit ZIP codes were mostly surrounded by rural areas. From 2018 to 2019, a stable High-High core cluster persisted in the 479 3-digit ZIP code, which means a high proportion of patients receiving an opioid prescription within the region and its immediate neighborhood, which are mostly urban areas (See Supplementary Material Table S4 and Table S5). Despite a decline in the rate of opioid prescriptions in 2018 and 2019, three urban areas—ZIP codes 461, 478, and 479— have some of the highest rates of opioid prescriptions per 1,000 population. In 2018, these areas reported between 129 and 175 opioid prescriptions per 1,000 population. In the following years, the rates decreased to around 70 to 106 prescriptions per 1,000 population (See Supplementary Material Table S6). Supplementary Material Table S5 provides comprehensive p-value information for each significant 3-digit ZIP code by year. It is crucial to clarify that the spatial autocorrelation method highlights the clusters or regions with a spatially concentrated proportion of patients receiving at least one opioid prescription. Areas labeled as “Not significant” imply that patients receiving opioid prescriptions in those regions are distributed randomly and have no significant connection to geospatial patterns; this, however, does not indicate the absence of opioid prescriptions.

### Evaluating the modifiable areal unit problem

To determine whether spatial aggregation influences the geographic patterns of opioid prescribing rates and the identification of high-exposure regions in Indiana, we evaluated the MAUP. This analysis aimed to determine whether the trends observed at the 3-digit ZIP code level in the proportion of patients receiving at least one opioid prescription remain consistent when aggregating to a different spatial unit. Information regarding the counties associated with each clustered 3-digit ZIP code, as well as the county-level Local Moran’s I results from 2015 to 2019 are presented in Supplementary Material Table S4 and Figure S7, respectively. A comparison between these spatial units revealed that, despite differences in spatial resolution, both levels of aggregation identify similar regional trends in the proportion of patients receiving at least one opioid prescription. Notably, the high-exposure areas in southwestern Indiana (3-digit ZIP codes 460, 476, 478, and 479) remain significant when converted to the county level, supporting the robustness of our findings. However, we found more spatial clusters of the proportion of patients prescribed at least one opioid prescription at the county level compared to the 3-digit ZIP code level. In contrast, there are finer spatial distinctions, such as the High-Low transitions in suburban regions (e.g., 3-digit ZIP code 472 in 2015), which become more apparent at the 3-digit ZIP code level. This pattern expands at the county level, leading to more homogenized spatial clusters, particularly due to the redistribution of patients prescribed at least one opioid prescription. This redistribution process reallocates the number of patients receiving at least one opioid prescription across various counties. We used residential address ratios from a 3-digit ZIP code within each county, based on data from the HUD crosswalk tables. This redistribution ensures that neighboring counties are statistically similar, which can artificially increase spatial autocorrelation. These subtle transitions emphasize the effects of the county-level aggregation, which tends to smooth out localized variations that are evident at the 3-digit ZIP code level. Moreover, counties might seem a significant cluster due to the inclusion of just one high-exposure 3-digit ZIP code. County-level aggregation redistributes data in a way that enhances the detectability of spatial autocorrelation, resulting in more pronounced clusters. These findings align with previous studies [27, 31, 35], which demonstrate that aggregation and spatial reallocation can artificially enhance spatial correlations due to the loss of local variations and the creation of artificial spatial clusters.

To quantitively assess the impact of MAUP, we ran an OLS bivariate regression analysis at the 3-digit ZIP code and county level, between the proportion of patients receiving at least one opioid prescription over time (See Supplementary Material Table S8). The measured $\:{\text{R}}^{2}$ is slightly low in the 3-digit ZIP code level ($\:{\text{R}}^{2}=0.4689$) compared to county-level ($\:{\text{R}}^{2}=0.5141$). The similarity in $\:{\text{R}}^{2}$ values across 3-digit ZIP codes and county level suggest that both spatial units capture the same underlying temporal trends in the number of patients receiving at least one opioid prescription. In both models, the coefficient for the proportion of patients receiving at least one opioid prescription was significant and implied a decline in opioid prescribing rates over time (3-digit ZIP code level = -0.0238, p-value < 0.001; county-level = -0.0259, p-value < 0.01). Moran’s I for OLS residuals was non-significant at the 3-digit ZIP code level, indicating no strong spatial dependency left that was not accounted for in the OLS model. However, Moran’s I for OLS residuals at the county level was significant. This indicates that the OLS model cannot account for spatial dependency at the county level. This could indicate that spatial dependencies may have been introduced during the county-level aggregation process.

## Discussion

This study examined the spatial and temporal patterns of opioid prescribing in rural and urban areas of Indiana, emphasizing the differences among population subgroups within a Medicaid beneficiary cohort using an analytical framework that combines data mining, statistics, and GIS techniques.

Specifically, our results indicate that older patients, who identify as White, females, and living in urban areas were more likely to receive at least one opioid prescription over time. Post-partum women are also more likely to be prescribed opioids for pain management following either a cesarean or vaginal delivery [36–39], which understandably increases the differences in opioid prescriptions compared to men [40, 41]. When examining the interactions between rural/urban classification and race/ethnicity, we found that patients from non-White racial backgrounds living in rural areas were less likely to receive at least one opioid prescription. Recent research has demonstrated that patterns of opioid overdoses reflect prescription rates, with majority-White and lower-income areas showing a greater likelihood of receiving at least one opioid prescription [42]. Furthermore, studies indicate a significantly higher rate of opioid prescriptions in areas characterized by high social vulnerability and deprivation as measured by the Social Vulnerability Index (SVI) and Area Depravation Index (ADI) [43]. These two composite indices measure the ability of the community to respond to external stresses, as well as, neighborhood-level disadvantage based on demographic factors such as income, and education, among others [44, 45]. High social vulnerability and deprivation are often found in rural regions [46]. Other studies have also shown disparities in race/ethnicity, where patients reported as White are more likely to receive opioid prescriptions [47, 48]. Additionally, in this study, we found that older patients tend to receive at least one opioid prescription, even after accounting for the interaction with other demographic variables. These populations may experience a higher frequency, longer duration, or greater intensity of chronic pain, which may contribute to a greater likelihood of receiving opioid prescriptions [49, 50].

Finally, although there was an increase in the number of patients receiving at least one opioid prescription from 2015 to 2016, likely due to the expansion of Medicaid that took effect in 2014 [51], it is important to note that there was a significant decline in prescriptions following 2016 (see Supplementary Material Table S2 and Table S6). Several studies evaluated the impact of Prescription Drug Monitoring Programs (PDMPs) in Indiana, indicating that the introduction of Public Law 194 significantly reduced opioid prescribing, particularly in vulnerable populations such as pregnant women and Medicaid enrollees [52–54]. After 2017, our analysis revealed that patients living in rural areas were less likely to receive opioid prescriptions compared to those in urban areas, coinciding with a decrease in the proportion of rural residents receiving prescriptions and an increase in urban areas. Additionally, the Global Moran’s I value increased until 2018, followed by a significant decrease in 2019, indicating that opioid prescribing was more widely distributed across Indiana. This implies that the decrease observed and the shift from rural to urban areas may be attributed to regulatory programs [55], although further analysis is necessary to confirm this impact. These regulations may lead patients from rural areas to seek healthcare facilities in more populated cities to obtain their opioid prescriptions, thus increasing the number of opioid prescribing rates in urban areas. Further studies are needed to assess the impact of healthcare facilities potentially becoming tertiary care centers for larger areas, which could contribute to an increase in the rate of opioid prescribing in urban areas. Additionally, further research is required to evaluate the effects of PDMPs in observed shifted hotspots of opioid prescribing rates.

The study does have certain limitations that should be acknowledged. Firstly, the spatial autocorrelation analysis relied on aggregated regions instead of precise 5-digit ZIP code, county-level, or census tract data, primarily due to privacy concerns limiting the availability of specific patient locations. Consequently, the behavior of clusters might differ when examined at more granular geographic scales. To mitigate this concern, we evaluated the MAUP by converting the number of patients receiving at least one opioid prescription at the 3-digit ZIP code to county-level estimates using HUD crosswalk tables. While this aggregation altered some spatial cluster distributions, the overall trends in opioid prescribing rates remained consistent, reinforcing the robustness of our findings at the 3-digit ZIP code level. However, future research should explore finer spatial resolutions when relevant information is accessible. Secondly, conducting a spatiotemporal analysis using the number of opioids dispensed may alter the results. However, we are limited in our demonstration due to the lack of information on the quantity of opioids dispensed with each prescription. Thirdly, our study cohort exclusively comprises patients covered by a single type of insurance, and generalizability should be approached with caution. Future research should incorporate diverse public and private insurance types to gain a more comprehensive perspective. Finally, using estimates of Medicaid enrollees from the ACS to normalize the number of patients receiving opioid prescriptions by 3-digit ZIP code may introduce bias. This could arise from factors such as self-reported enrollment, uncertainty in smaller areas, and the inability to verify whether the population in our cohort aligns with the Medicaid enrollees from the ACS.

This study offers crucial insights into evaluating opioid prescribing patterns in rural and urban areas through spatiotemporal analysis to map geographical trends and identify potential differences in opioid prescribing rates. This approach can be replicated with more localized data, revealing important aspects of opioid prescription behavior. Future research should concentrate on the behaviors of individual providers, specifically addressing the reasons behind the continued presence of outliers in prescribing practices. Additionally, future research should assess the impact of regulatory programs in reducing opioid prescribing risks, particularly in urban versus rural communities. There is still a need to enhance awareness regarding messaging and regulatory initiatives among rural providers. Furthermore, future studies should utilize spatial autocorrelation techniques in smaller and more specific areas, such as the 5-digit ZIP code level or census tracts/block groups level, to gain a better understanding of the dynamics of opioid prescribing with more accurate data.

## Conclusions

This study provides critical insights into the spatial and temporal patterns of opioid prescribing among Medicaid enrollees in Indiana, highlighting disparities between rural and urban areas and across demographic subgroups. Our findings emphasize significant shifts in opioid prescription rates from rural to urban areas over the study period, likely influenced by regulatory interventions such as PDMPs. These results underscore the importance of targeted strategies to address geographic and demographic disparities in opioid prescribing while accounting for the evolving role of healthcare access and regulatory impacts. Future research should explore more granular geographic levels, diverse insurance cohorts, and provider-specific behaviors to further understand the dynamics of opioid prescribing and inform effective public health interventions.

## Electronic supplementary material

Below is the link to the electronic supplementary material.

**Supplementary Material Table S1.** Prescription Opioids extracted from the National Drug Code Directory, US Food & Drug Administration. **Supplementary Material Table S2.**Negative binomial regression results.**Supplementary Material Table S3.**Global Moran’s I and its respective p-value each year. Global Moran’s I and its respective p-value each year. **Supplementary Material Table S4.**Counties assigned to each 3-digit ZIP codes and their RUCA classification. **Supplementary Material Table S5.**Local Moran’s I for the most significant areas by year. **Supplementary Material Table S6.**The rate of opioid prescriptions by 1,000 for each 3-digit ZIP code across years. **Supplementary Material Figure S7.**Spatial clusters with normalized patients receiving an opioid prescription at the county-level, Indiana, 2015–2019. **Supplementary Material Table S8.**OLS regression results for opioid prescribing rates over time.

## Data Availability

No datasets were generated or analysed during the current study.

## Abbreviations

GIS: Geographic information systems
FSSA: Indiana Family and Social Services Administration
NDC: National Drug Code directory
NPI: Billing National Provider Identifiers
RUCA: Rural-Urban Commuting Area Codes
ACS: American Community Survey
LISA: Local Indicators of Spatial Autocorrelation
MAUP: Modifiable areal unit problem
PDMPs: Prescription Drug Monitoring Programs
