# Supplementary material for: A spatiotemporal analysis of opioid prescriptions in Indiana from 2015 to 2019
Source: Subst Abuse Treat Prev Policy. 2025 Aug 8;20:30. doi: 10.1186/s13011-025-00664-8 (PMC12335077; doi:10.1186/s13011-025-00664-8)
Supplement: Supplementary file 1 — Supplementary Material Table S1. Prescription Opioids extracted from the National Drug Code Directory, US Food & Drug Administration. Supplementary Material Table S2.Negative binomial regression results.Supplementary Material Table S3.Global Moran’s I and its respective p-value each year. Global Moran’s I and its respective p-value each year. Supplementary Material Table S4.Counties assigned to each 3-digit ZIP codes and their RUCA classification. Supplementary Material Table S5.Local Moran’s I for the most significant areas by year. Supplementary Material Table S6.The rate of opioid prescriptions by 1,000 for each 3-digit ZIP code across years. Supplementary Material Figure S7.Spatial clusters with normalized patients receiving an opioid prescription at the county-level, Indiana, 2015–2019. Supplementary Material Table S8.OLS regression results for opioid prescribing rates over time. [file 13011_2025_664_MOESM1_ESM.docx]

**Supplementary Material**

**Supplementary Material Table S1.** Prescription Opioids extracted from the National Drug Code Directory, US Food & Drug Administration*

| Fentanyl Citrate |
| --- |
| Hydromorphone Hydrochloride |
| Morphine Sulfate |
| Meperidine Hydrochloride |
| Hydrocodone Bitartrate; Acetaminophen |
| Oxycodone Hydrochloride; Acetaminophen |
| Oxycodone Hydrochloride |
| Methadone Hydrochloride |
| Fentanyl |
| Buprenorphine Hydrochloride; Naloxone Hydrochloride |
| Hydrocodone Bitartrate; Ibuprofen |
| Buprenorphine Hydrochloride; Naloxone Hydrochloride Dihydrate |
| Buprenorphine Hydrochloride |
| Hydrocodone Bitartrate; Homatropine Methylbromide |
| Acetaminophen; Hydrocodone Bitartrate |
| Buprenorphine |
| Buprenorphine Hydrochloride; Naloxone |
| Hydrocodone Bitartrate; Chlorpheniramine Maleate |
| Acetaminophen; Hydrocodone Bitartrate |
| Buprenorphine |
| Buprenorphine Hydrochloride; Naloxone |
| Hydrocodone Bitartrate; Chlorpheniramine Maleate |

*All the NDC codes are in a list submitted in the GitHub folder (OpioidNDC.xlsx)

**Supplementary Material Table S2.** Negative binomial regression results

| **Variable** | **Rate ratio** | **Std error** | **z-value** | **p-value** | **95% CI -Lower** | **95% CI -Upper** |
| --- | --- | --- | --- | --- | --- | --- |
| (Intercept) | 3350.349 | 0.142 | 57.02 | 0.000 | 2531.041 | 4467.994 |
| Year | 0.703 | 0.038 | -9.307 | 0.000 | 0.651 | 0.760 |
| **Age** |  |  |  |  |  |  |
| Age 18 to 25 | Ref |  |  |  |  |  |
| Age 26 to 34 | 1.453 | 0.159 | 2.356 | 0.018 | 1.062 | 1.987 |
| Age 35 to 44 | 1.710 | 0.158 | 3.392 | 0.001 | 1.249 | 2.341 |
| Age 45 to 54 | 1.830 | 0.159 | 3.805 | 0.000 | 1.333 | 2.511 |
| Age 55 to 64 | 1.523 | 0.159 | 2.642 | 0.008 | 1.106 | 2.098 |
| **Race** |  |  |  |  |  |  |
| Race White | Ref |  |  |  |  |  |
| Race Black | 0.389 | 0.147 | -6.406 | 0.000 | 0.291 | 0.522 |
| Race Hispanic | 0.096 | 0.159 | -14.714 | 0.000 | 0.070 | 0.132 |
| Race Other | 0.009 | 0.178 | -26.452 | 0.000 | 0.006 | 0.013 |
| **Sex** |  |  |  |  |  |  |
| Female | Ref |  |  |  |  |  |
| Male | 0.277 | 0.134 | -9.540 | 0.000 | 0.212 | 0.363 |
| **Rural/Urban** |  |  |  |  |  |  |
| Urban | Ref |  |  |  |  |  |
| Rural | 0.249 | 0.124 | -11.242 | 0.000 | 0.195 | 0.319 |
| **Interaction effects** |  |  |  |  |  |  |
| Year × Age 26 to 34 | 1.118 | 0.039 | 2.854 | 0.004 | 1.032 | 1.211 |
| Year × Age 35 to 44 | 1.076 | 0.039 | 1.881 | 0.060 | 0.994 | 1.165 |
| Year × Age 45 to 54 | 1.039 | 0.039 | 0.978 | 0.328 | 0.960 | 1.125 |
| Year × Age 55 to 64 | 1.026 | 0.039 | 0.646 | 0.519 | 0.947 | 1.111 |
| Year × Race Black | 0.965 | 0.031 | -1.131 | 0.258 | 0.907 | 1.028 |
| Year × Race Hispanic | 1.008 | 0.034 | 0.230 | 0.818 | 0.941 | 1.079 |
| Year × Race Other | 1.353 | 0.038 | 7.993 | 0.000 | 1.252 | 1.462 |
| Age 26 to 34 × Race Black | 0.775 | 0.140 | -1.827 | 0.068 | 0.589 | 1.019 |
| Age 35 to 44 × Race Black | 0.689 | 0.139 | -2.671 | 0.008 | 0.524 | 0.906 |
| Age 45 to 54 × Race Black | 0.720 | 0.139 | -2.370 | 0.018 | 0.548 | 0.945 |
| Age 55 to 64 × Race Black | 0.768 | 0.139 | -1.898 | 0.058 | 0.584 | 1.008 |
| Age 26 to 34 × Race Hispanic | 0.765 | 0.151 | -1.773 | 0.076 | 0.568 | 1.031 |
| Age 35 to 44 × Race Hispanic | 0.663 | 0.150 | -2.727 | 0.006 | 0.494 | 0.891 |
| Age 45 to 54 × Race Hispanic | 0.553 | 0.151 | -3.914 | 0.000 | 0.411 | 0.745 |
| Age 55 to 64 × Race Hispanic | 0.518 | 0.152 | -4.312 | 0.000 | 0.384 | 0.700 |
| Age 26 to 34 × Race Other | 0.890 | 0.169 | -0.688 | 0.492 | 0.640 | 1.240 |
| Age 35 to 44 × Race Other | 0.785 | 0.168 | -1.444 | 0.149 | 0.564 | 1.092 |
| Age 45 to 54 × Race Other | 0.500 | 0.172 | -4.031 | 0.000 | 0.356 | 0.702 |
| Age 55 to 64 × Race Other | 0.552 | 0.173 | -3.437 | 0.001 | 0.392 | 0.777 |
| Year × Male | 1.052 | 0.031 | 1.639 | 0.101 | 0.988 | 1.120 |
| Age 26 to 34 × Male | 0.969 | 0.111 | -0.285 | 0.776 | 0.779 | 1.204 |
| Age 35 to 44 × Male | 1.285 | 0.110 | 2.271 | 0.023 | 1.035 | 1.595 |
| Age 45 to 54 × Male | 1.761 | 0.111 | 5.104 | 0.000 | 1.416 | 2.189 |
| Age 55 to 64 × Male | 1.874 | 0.111 | 5.643 | 0.000 | 1.506 | 2.333 |
| Race Black × Male | 1.118 | 0.088 | 1.268 | 0.205 | 0.941 | 1.327 |
| Race Hispanic × Male | 0.936 | 0.097 | -0.683 | 0.495 | 0.774 | 1.132 |
| Race Other × Male | 1.572 | 0.108 | 4.189 | 0.000 | 1.272 | 1.943 |
| Year × Rural | 1.004 | 0.035 | 0.111 | 0.911 | 0.936 | 1.076 |
| Race Black × Rural | 0.196 | 0.089 | -18.342 | 0.000 | 0.165 | 0.233 |
| Race Hispanic × Rural | 0.265 | 0.102 | -12.969 | 0.000 | 0.217 | 0.324 |
| Race Other × Rural | 0.426 | 0.123 | -6.933 | 0.000 | 0.333 | 0.541 |
| Male × Rural | 1.432 | 0.164 | 2.194 | 0.028 | 1.031 | 1.989 |
| Year × Male × Rural | 0.909 | 0.051 | -1.882 | 0.060 | 0.821 | 1.007 |

*****The rate ratio was derived by calculating the exponential of the log counts from the model.

**Supplementary Material Table S3.** Global Moran’s I and its respective p-value each year.

| **Year** | **Global Moran's I** |
| --- | --- |
| 2015 | 0.0098 * |
| 2016 | 0.0544 * |
| 2017 | 0.1706 * |
| 2018 | 0.2550 ** |
| 2019 | 0.1768 * |

* p-value > 0.05, ** p-value <0.05

**Supplementary Material Table S4.** Counties assigned to each 3-digit ZIP codes and their RUCA classification.

| **3-digit ZIP codes** | **Counties assigned*** | **RUCA classification**** |
| --- | --- | --- |
| 460 | Boone County, Clinton County, Hamilton County, Madison County, Tipton County | Urban |
| 461 | Hancock County, Hendricks County, Johnson County, Morgan County, Putnam County, Rush County, Shelby County | Urban |
| 462 | Marion County | Urban |
| 463 | LaPorte County, Porter County | Urban |
| 464 | Lake County | Urban |
| 465 | Elkhart County, Kosciusko County, Marshall County, Starke County | Urban |
| 466 | St. Joseph County | Urban |
| 467 | Adams County, DeKalb County, Huntington County, LaGrange County, Noble County, Steuben County, Wells County, Whitley County | Rural |
| 468 | Allen County | Urban |
| 469 | Carroll County, Cass County, Fulton County, Grant County, Howard County, Miami County, Pulaski County, Wabash County | Rural |
| 470 | Dearborn County, Franklin County, Ohio County, Ripley County, Switzerland County | Rural |
| 471 | Clark County, Crawford County, Floyd County, Harrison County, Scott County, Washington County | Urban |
| 472 | Bartholomew County, Decatur County, Jackson County, Jefferson County, Jennings County | Urban |
| 473 | Blackford County, Delaware County, Fayette County, Henry County, Jay County, Randolph County, Union County, Wayne County | Rural |
| 474 | Brown County, Greene County, Lawrence County, Monroe County, Orange County, Owen County | Urban |
| 475 | Daviess County, Dubois County, Knox County, Martin County, Perry County, Pike County | Rural |
| 476 | Gibson County, Posey County, Spencer County, Warrick County | Urban |
| 477 | Vanderburgh County | Urban |
| 478 | Clay County, Parke County, Sullivan County, Vermillion County, Vigo County | Urban |
| 479 | Benton County, Fountain County, Jasper County, Montgomery County, Newton County, Tippecanoe County, Warren County, White County | Urban |

*Counties were assigned to each 3-digit ZIP code using the ZIP Code Tabulation Area (ZCTA) to County relationship files provided by the U.S. Census Bureau. We aggregated land area estimates by the first 3-digits of the ZCTA. If a county was included in multiple 3-digit ZIP codes, it was assigned to the ZCTA code covering the largest land area. When a 3-digit ZIP code covered only one county, that county was directly assigned to it.

**The RUCA by 3-digit ZIP code was calculated as a weighted average using the equation as follows:

$$RUCA_{3-digit}=\frac{\sum RUCA_{5-digit}*Population_{5-digit}}{\sum Population_{5-digit}}$$

Where $RUCA_{5-digit}$ is the RUCA Codes assigned to each 5-digit ZIP code, $Population_{5-digit}$ is the total population of individuals aged 18 to 64 from each 5-digit ZIP code provided by the U.S. Census Bureau.

**Supplementary Material Table S5.** Local Moran’s I for the most significant areas by year.

| **Year** | **Most significant areas** | **Nearest most populated cities**** | **Local Moran's I *** |
| --- | --- | --- | --- |
| 2015 | 472 | Bloomington | -1.002 |
| 2016 | 472 | Bloomington | -0.995 |
|  | 474 | Bloomington | -0.6889 |
|  | 476 | Evansville | 1.108 |
| 2017 | 460 | Carmel, Noblesville, Fishers, Indianapolis, Lafayette | -0.099 |
|  | 478 | Lafayette, Bloomington | 0.158 |
| 2018 | 479 | Hammond, Lafayette, Carmel, Noblesville, Fishers | 1.064 |
| 2019 | 478 | Lafayette, Bloomington | 1.237 |
|  | 479 | Hammond, Lafayette, Carmel, Noblesville, Fishers | 1.303 |

* p-value <0.05. ** These cities are in the top 10 of most populated cities in Indiana.

**Supplementary Material Table S6.** The rate of opioid prescriptions by 1,000 for each 3-digit ZIP code across years.

| **3-digit ZIP code** | **Rate of opioid prescription by 1,000*** | | | | |
| --- | --- | --- | --- | --- | --- |
|  | **2015** | **2016** | **2017** | **2018** | **2019** |
| 460 | 223.505 | 277.210 | 56.948 | 78.409 | 42.410 |
| 461 | 182.571 | 245.537 | 137.943 | 129.112 | 70.649 |
| 462 | 150.509 | 212.982 | 133.291 | 104.196 | 53.074 |
| 463 | 185.724 | 233.946 | 72.186 | 121.953 | 41.774 |
| 464 | 141.873 | 157.574 | 66.064 | 103.324 | 28.154 |
| 465 | 139.385 | 179.563 | 29.359 | 46.392 | 27.345 |
| 466 | 51.029 | 77.045 | 9.841 | 11.148 | 2.256 |
| 467 | 189.323 | 258.608 | 57.018 | 97.639 | 45.462 |
| 468 | 189.188 | 271.408 | 82.854 | 111.419 | 32.827 |
| 469 | 135.584 | 198.617 | 67.525 | 77.231 | 47.364 |
| 470 | 42.187 | 54.163 | 26.336 | 36.606 | 35.330 |
| 471 | 129.554 | 213.590 | 55.402 | 63.225 | 32.301 |
| 472 | 228.892 | 309.498 | 53.687 | 47.853 | 27.036 |
| 473 | 134.803 | 203.956 | 47.039 | 45.254 | 21.177 |
| 474 | 103.294 | 137.557 | 25.420 | 29.924 | 18.789 |
| 475 | 190.775 | 391.284 | 96.609 | 97.932 | 43.081 |
| 476 | 236.872 | 335.511 | 81.615 | 95.688 | 31.408 |
| 477 | 272.112 | 343.337 | 51.287 | 69.187 | 21.554 |
| 478 | 235.105 | 264.386 | 60.374 | 69.788 | 106.523 |
| 479 | 201.750 | 293.338 | 140.698 | 175.943 | 80.794 |

*The rate of opioid prescriptions was calculated by counting the number of opioid prescriptions and dividing it by the total of patients receiving at least one opioid prescription from our cohort by year. This process was made for each of the 3-digit ZIP codes.

**Supplementary Material Figure S7.** Spatial clusters with normalized patients receiving an opioid prescription at the county-level, Indiana, 2015–2019*


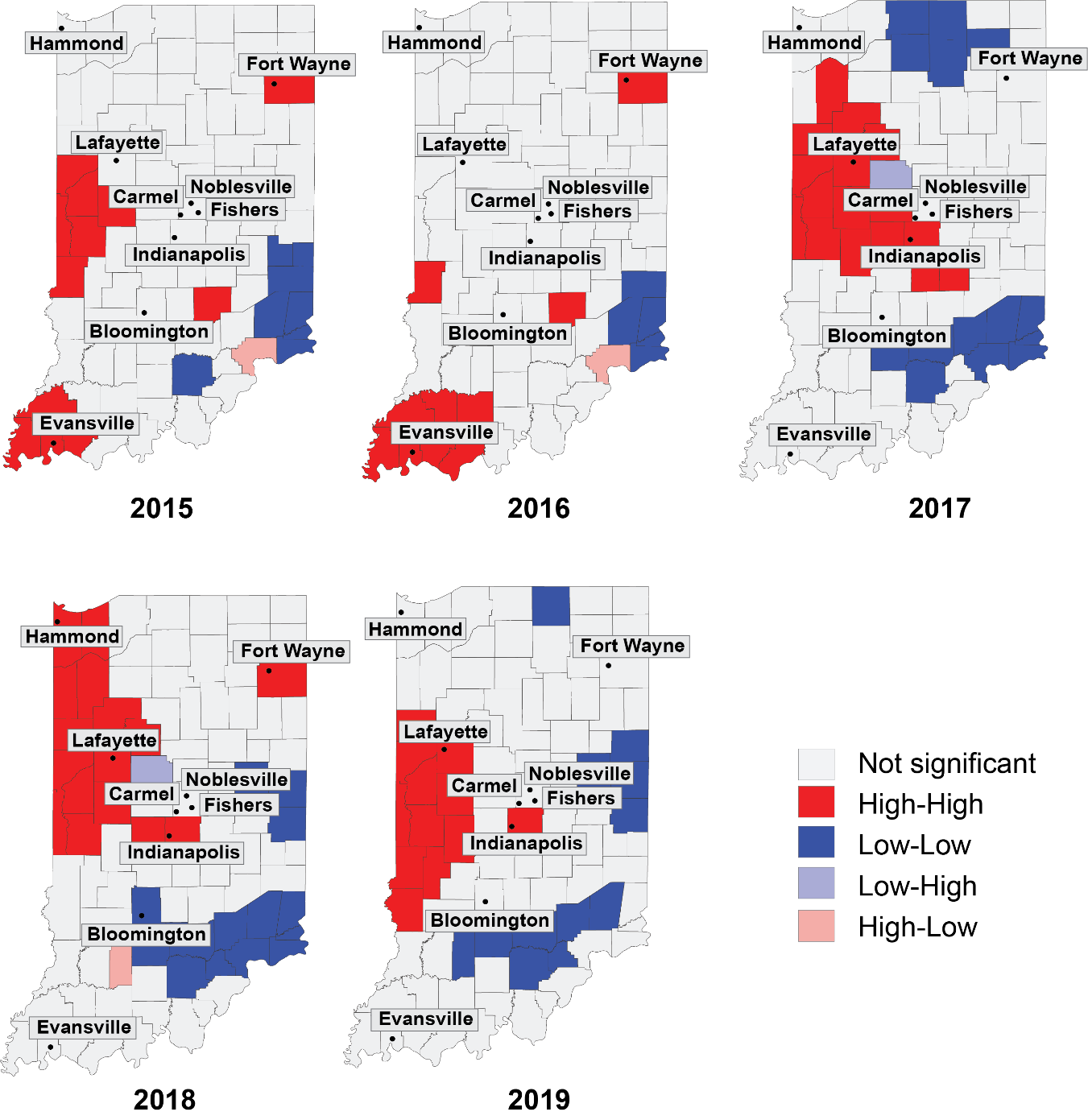


*We converted the number of patients taking at least one opioid prescription from the 3-digit ZIP code level to the county level using the crosswalks from the U.S. Department of Housing and Development (HUD). We first obtained the weighted redistribution as follows:

$$O_{county}=O_{ZIP3}xResRatio$$

Where $O_{ZIP3}$ is the estimated number of individuals taking at least one opioid prescription and $ResRatio$ is the total ratio of the 3-digit ZIP code population that is within each county. The redistributed number of individuals taking at least one opioid prescription was summed across all 3-digit ZIP codes within each county. Finally, the redistributed number of patients taking at least one opioid prescription was divided by the total Indiana Medicaid population from ACS at the county level to obtain the proportion of patients taking at least one opioid prescription.

**Supplementary Material Table S8.** OLS regression results for opioid prescribing rates over time

| **Variable** | **3-Digit ZIP code level** | **County-level** |
| --- | --- | --- |
| Intercept | 2018.0 (0.185)*** | 2019.0 (0.087)*** |
| Opioid prescription prescribing rate coefficient | -0.0238 (0.0025)*** | -0.0259 (0.0012)*** |
| Adjusted $R^{2}$ | 0.4698 | 0.5141 |
| F-statistic | 88.71 | 486.7 |
| Residual Std. Error | 1.035 | 0.9868 |
| Moran’s I (Residuals) | 0.0138 (p-value = 0.2083) | 0.0723 (p-value < 0.001)*** |
| Jarque-Bera test | 3.8596 (p-value = 0.1452) | 17.41 (p-value <0.001) *** |
| Breusch-Pagan test | 1.315 (p-value = 0.2515) | 2.0307 (p-value = 0.1542) |

Standard errors are in parentheses. ***p-value < 0.001; **p-value < 0.01; *p-value < 0.05.
